# Biodiversity of Genetic, Metabolic, and Antibiotic Resistance Profiles of *Escherichia coli* Strains Recovered from the Baltic Sea Region

**DOI:** 10.3390/microorganisms14061212

**Published:** 2026-05-27

**Authors:** Marta Potrykus, Monika Kurpas, Anna Sawik, Anna Budzyńska, Krzysztof Skowron

**Affiliations:** 1 Laboratory of Biologically Active Compounds, Intercollegiate Faculty of Biotechnology University of Gdansk and Medical University of Gdansk, University of Gdansk, Abrahama 58, 80-307 Gdansk, Poland; 2 Department of Immunobiology and Environmental Microbiology, Faculty of Health Sciences with the Institute of Maritime and Tropical Medicine, Medical University of Gdansk, Debinki 7, 80-204 Gdansk, Poland; monika.kurpas@gumed.edu.pl; 3 Department of Microbiology, Ludwik Rydygier Collegium Medicum in Bydgoszcz, Nicolaus Copernicus University in Toruń, 9 M. Skłodowska-Curie St., 85-094 Bydgoszcz, Poland; a.budzynska@cm.umk.pl (A.B.); krzysztof.skowron@cm.umk.pl (K.S.)

**Keywords:** antibiotic resistance, rep-PCR, *E. coli* identification, marine environment

## Abstract

Marine environments represent a significant reservoir that facilitates the spread of antibiotic resistance. In the present study, 69 samples (seawater, sand, and surface water) were collected from 16 points of high anthropogenic pressure along the Baltic Sea in 2022–2023. Out of these, 184 strains of lactose-fermenting bacteria were isolated, and 24 species were identified by MALDI TOF-MS, with *Escherichia coli* being the most prevalent (114 strains). Bacterial isolates were assigned to phylogroups and profiled with repetitive sequence PCR and API and evaluated for susceptibility to 16 antibiotics: 61% of *E. coli* strains belonged to commensal/environmental B1 and A phylogroups, while 20% belonged to potentially pathogenic B2 or D phylogroups. Moreover, 18% of the isolates exhibited resistance to at least one antibiotic, while seven were multidrug-resistant. For amikacin and gentamicin, strains from the B2 and D phylogroups had significantly smaller average growth inhibition zones than strains from the A and B1 phylogroups. Moreover, for six antibiotics belonging to each WHO AWaRe group (Access, Watch and Reserve), average growth inhibition zone diameters were significantly smaller in samples collected in warmer months vs. those obtained in colder months. The results suggest that monitoring for antibiotic-resistant strains should extend from recreational waters to beach sands, prioritizing summer sampling over winter.

## 1. Introduction

The Baltic Sea (Europe) is one of the few shallow brackish (approx. 7‰ salinity) water reservoirs worldwide. Thus, it represents a unique environment for the growth and survival of microorganisms [1,2]. In fact, the diversity of bacterial communities in the Baltic Sea is more enriched than in most other ocean environments because species that are better adapted to low salinity can persist in the Baltic Sea marine environment [2]. However, at the same time, the microbial diversity of the Baltic Sea has also been negatively influenced by heavy anthropogenic pressure, because the Baltic Sea catchment basin (1,729,500 km^2^) is inhabited by over 85 million people from nine different countries [3]. Based on reports from the Convention on the Protection of the Marine Environment of the Baltic Sea Area (HELCOM), most of the monitored locations are categorized as ‘moderate-to-poor’ with respect to the level of chemical contamination. In particular, pharmaceutical compound contaminants have been detected in the Baltic Sea ecosystem and in effluents discharged from 46 wastewater treatment plants located along the Baltic Sea catchment basin [4]. Thus, this constant pressure from antibiotic compounds contamination may play a crucial role in shaping and enabling the acquisition of microbial resistance in this marine environment [5]. In 2021, the World Health Organization proposed the AWaRe classification scheme in order to encourage more responsible use of antibiotics. The AWaRe classification divides antibiotics into Access, Watch and Reserve groups, based on the risk of developing antibiotic resistance to these drugs and their relevance to medicine [6]. In the Baltic Sea area in general, the average use of antimicrobial drugs was reported to be 25% lower than the EU average (15.39 DDD per 1000 inhabitants per day) [7]. However, in 2023, Poland, Lithuania and Denmark all reported higher than average antibiotic use rates, corresponding to 23.2, 18.7 and 16.2 DDD per 1000 inhabitants per day, respectively. Interestingly, in Denmark, 80% of these prescribed antimicrobial drugs have been categorized as access antibiotics, while in Poland, only 60.4% prescriptions belonged to this class. Also, the concentration of antimicrobial compounds in coastal waters of the Baltic Sea was found to be lower than in inland surface waters, such as rivers [8]. Nevertheless, the most frequently assessed and detected antibiotics in water and/or sediment samples from the Baltic Sea were trimethoprim, sulfamethoxazole, tetracycline (AWaRe Access group), oxytetracycline (AWaRe Watch group), and enrofloxacin (not classified) [9,10,11,12,13,14]. Strikingly, multidrug-resistant *E. coli* strains have even been found to occur along an antibiotic residue gradient in the coastal waters of the Baltic Sea, as verified by the concentration levels of ampicillin, ciprofloxacin, and sulfamethoxazole measured at sites where extended-spectrum beta-lactamase (ESBL)-producing *E. coli* were isolated [5]. *E. coli* is a rod-shaped, Gram-negative bacterium from the Enterobacteriaceae family, which inhabits the intestinal tract of healthy humans [15]. Concurrently, *E. coli* remains the benchmark indicator for tracking fecal contamination in recreational waters. Various strategies exist for their detection in environmental samples, ranging from conventional cultivation on selective media and the use of fluorescently labeled sera to the enzymatic assays of beta-glucuronidase activity [16]. Crucially, *E. coli*, rather than being transient microbial contaminants introduced into the environment mainly via sewage, can survive long-term and become naturalized within novel habitats such as waterways [17]. Differentiating between enteric and naturalized *E. coli* fractions remains a significant challenge. Furthermore, it is not clear whether these naturalized populations can still efficiently infect warm-blooded animals, including humans. Nevertheless, they still can act as environmental reservoirs for antimicrobial resistance and virulence genes [18]. However, in some instances, *E. coli* can also cause diseases, including serious bloodstream and urinary tract infections [6]. In fact, *E. coli* was included among the top 12 antibiotic-resistant pathogens by the WHO (https://www.who.int/publications/i/item/WHO-EMP-IAU-2017.12 (accessed on 2 August 2025)). Moreover, in 2023, *E. coli* was the most frequently reported cause of bloodstream infections and urinary tract infections globally and is responsible for nearly half of all reported infections [6]. Unfortunately, treatment of these *E. coli* infections with Access group antibiotics is limited, and the resistance levels of *E. coli* to Watch group antibiotics are diverse. For example, the percentage resistance to ciprofloxacin was reported to be 40.7%, while resistance to third-generation cephalosporins was found to be between 43.5% (cefotaxime) and 31.8% (ceftazidime), depending on the antibiotic type, which, in general, is often indicative of ESBL-production [6].

The marine environment represents a reservoir of natural and multi-drug-resistant (MDR) strains of *E. coli*, and wastewater treatment plants can harbor a significant amount of antibiotic-resistant microorganisms, which are then discharged into marine waters [19,20]. Antibiotic-resistant *E. coli* strains isolated from waste waters are most commonly resistant to amoxicillin/clavulanate (32%), trimethoprim/sulfamethoxazole (20%), and fluoroquinolone (15%) [21]. In addition, in samples obtained from Baltic Sea mammals (66 samples), drug-resistant *E. coli* strains were also found, among which 37.2% were MDR [22]. Interestingly, in these samples, strains displayed resistance primarily against aminoglycosides and penicillins. It is also worth noting that the surrounding areas of the specific collection sites in the Baltic Sea may strongly impact the prevalence of drug-resistant *E. coli* [5]. For example, among 124 samples collected along the German coast between 2021 and 2022, 30 *E. coli* strains were found to be ESBL-positive. These strains were primarily isolated from samples obtained near a wastewater treatment plant (60%) and an urban beach (36.7%), while only one sample containing an ESBL-positive strain was collected near the rural area of Riems. In water reservoirs located by the shores of the Gulf of Gdansk (in the Baltic Sea), ESBL-producing strains of *E. coli* were isolated that were mostly resistant to cefuroxime and cefepime (100%), cefotaxime (approx. 90%), ceftazidime, ciprofloxacin, and sulfamethoxazole/trimethoprim (approx. 60%) [23]. Moreover, anthropogenic sources of contamination may in some cases lead to differentiation of drug-resistant patterns, as was shown for *E. coli* strains collected from three lakes subjected to different levels of anthropogenic pressure [24]. Jang et al. (2017) have shown that a thorough analysis of the presence of this pathogen and its survival in these environments may help us to assess the risk of acquiring MDR *E. coli* infections from recreational activities, such as bathing and sunbathing in marine surroundings [18].

In the present study, we aimed to assess the prevalence of MDR strains in bathing areas along the Gulf of Gdansk (Baltic Sea, Poland), which is under high anthropogenic pressure both in the local water and sandy beaches along the coast. Because *E. coli* isolates were the most abundant among the identified lactose-fermenting strains, antibiotic resistance, ESBL-production and blaCTX-M gene presence were evaluated for these strains to reveal the structure of the *E. coli* population in the Gulf of Gdansk and its surroundings. Moreover, the genetic and metabolic biodiversity of the collected *E. coli* strains was assessed to reveal potential correlations between the genotypes and phenotypes of these environmental isolates.

## 2. Materials and Methods

### 2.1. Samples Collection

**Figure 1:**
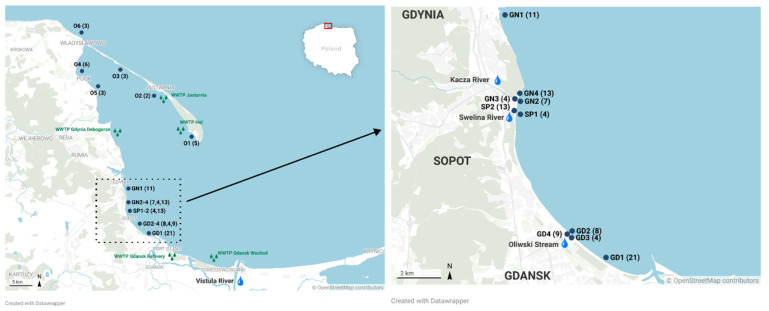
Sampling sites, from which samples were collected between 2022 and 2023. Samples were collected from 16 sites (4 in Gdansk (GD), 4 in Gdynia (GN), 2 in Sopot (SP) and 6 in other locations (O1-6)) along the Gulf of Gdansk, Baltic Sea, Poland, Europe. For details, see Table 1. The number of *E. coli* isolates from each location was marked in the brackets. WWTP—waste water treatment plants locations.

Samples were collected from recreational sandy beaches situated along the shores of the Gulf of Gdansk and Hel Peninsula, by the Baltic Sea southern shore, in Poland (Figure 1). For the Gulf of Gdansk, major natural water inflows are from precipitation (approx. 8 × 10^9^ m^3^/year), and the largest Polish river—Vistula (approx. 3.3 × 10^10^ m^3^/year); followed by several smaller rivers and streams, which add approx. 2.9 × 10^8^ m^3^/year (Table S3). In addition, there are 5 major wastewater treatment plants, which discharge sewage into the Gulf of Gdansk (approx. 6.13 × 10^7^ m^3^/year), mostly at a distance of 1.26 to 2.5 km from the shore (Table S3). Four of these treatment plants are mechanical-biological types, with chemical support for phosphorus removal, which do not exceed set norms for raw sewage parameters. One of these is a wastewater treatment plant that belongs to a refinery, which is the largest industrial company that discharges sewage into the Gulf of Gdansk. Moreover, there are two operational seaports (Gdansk, Gdynia) in the Gulf of Gdansk, which also add to the contamination of the area. A total of 69 samples (water and/or sand) were collected between 2022 and 2023 in 16 distinct locations (Figure 1 and Table 1). Samples from Hel Peninsula (O1–O6) were collected twice (14 June 2022 and 11 January 2023), while samples from the recreational beaches near Gdansk (GD1, GD2, GD3, GD4), Gdynia (GN1, GN2, GN3, GN4) and Sopot (SP1, SP2) were collected several times throughout the year (Table 1). Sand and water samples were collected following a protocol described by Kurpas et al. (2021), and sand samples were collected either from the shoreline, where the sand is constantly mixed with seawater (intertidal zone), or from areas adjacent to stream banks, near their mouth (GD4, GN4, SP2) [25]. Water samples were collected 1 m from the shore of the sea/river, 10–15 cm below the water surface. Samples were collected in the morning using sterile 50 mL Falcon tubes (for sand) (Sarstedt, Germany) and plastic 1 L sterile containers (for water) and were transferred directly to the laboratory. Upon arrival, the samples were processed within 4–6 h.

### 2.2. Isolation of Lactose-Fermenting Bacteria from Sand and Water Samples

For sand samples, 37.5 g of the wet sand was weighed aseptically and placed in a flask with 0.3 L of sterile distilled water. The mixture was then incubated for 1 h with shaking (250 rpm) at room temperature, to enable bacteria to detach from the sand particles. Then, the membrane filtration method was employed to isolate bacteria. Briefly, 20 mL of seawater, or 10 mL of the water obtained from the sand extraction, was passed through a sterile filter (0.45 µm pore diameter, Merck, Darmstadt, Germany) with the use of a Merck Millipore membrane filtration set. Then, the filter was placed on a MacConkey agar plate (Graso Biotech, Janowo, Poland) and incubated for 24 h at 37 °C. Up to eight lactose-fermenting colonies (if present) were then purified and stored as pure cultures in 20% glycerol stocks at −80 °C. The colonies were randomly selected and the number of selected colonies reflected the total number of colonies grown on the filter.

### 2.3. Species Identification

Species identification was performed by MALDI-TOF MS mass spectrometry using a Microflex LT/SH mass spectrometer from Bruker (Billerica, MA, USA), and the MALDI Biotyper software package (version 4.1). The Bruker Taxonomy reference database (Bruker, Billerica, MA, USA) and default parameter settings were used following the procedure described by Schulthess et al. (2013) [26]. For all tested strains, the score index level was ≥2.00, compared to reference spectra in the database, which enabled classification of the tested strains into different species.

Simultaneously, strains that were identified as *E. coli* in MALDI-TOF MS analysis were also subjected to API testing with the API20E kit for Enterobacteriaceae (Biomerieux, Marcy-l’Étoile, France).

First, the isolates were identified by MALDI-TOF MS, then subjected to API testing. In case of divergent results, some of the isolates were subjected to repeat MALDI-TOF MS identification, and in case of suspicious isolates, they were excluded from further analysis (Figure S3).

### 2.4. DNA Fingerprinting

Genomic DNA was isolated from pure bacterial cultures using the Genomic Mini AX Bacteria Kit (A&A Biotechnology, Gdańsk, Poland). The concentration and purity of the DNA were measured with a Nanodrop 1000 (Thermo Scientific, Waltham, MA, USA). Then, *E. coli* were analyzed using a repetitive-sequence-based rep-PCR with ERIC (ERIC 1F, ERIC 2R) and BOX (BOX 1) primers as described in Rybak et al. (2021) and Versalovic et al. (1998) [23,27]. The PCR reactions included 50 ng of bacterial DNA, 1× Thermofisher PCR buffer, 3.5 mM MgCl_2_, 4 µM of the primer(s), 1U of Taq Polymerase (Thermofisher), and 0.1 mM dNTPs (Nzytech, Lisboa, Portugal). After PCR, 5 µL of the products were resolved by 0.8% agarose gel electrophoresis (0.5× TBE) at 50 V for 135 min. Gels were stained with ethidium bromide solution (5 µL/100 mL H_2_O; Sigma Aldrich, St. Louis, MO, USA) for 20 min and visualized with the GelDoc (Biorad, Hercules, CA, USA) imaging system. The resulting band patterns were analyzed with GelJ software v2 [28] to uncover any relationships between the profiles. Trees were built using Pearson correlation as a similarity method, and Unweighted Pair Group Method with Arithmetic Mean (UPGMA) as a linkage method with a set tolerance of 1.0. The resulting distance matrix was used to draw a tree with the ape library version 5.8-1 in R 3.2.0.

### 2.5. Phylogroup Assignment of the E. coli Strains

Phylogroup assignment was performed as described in Clermont et al. (2013 and 2021), based on the determination of specific DNA gene fragments (*chuA*, *yjaA*, TSPE4.C2, *arpA* and *trpA*) for the *E. coli* strains [29,30]. PCR reactions were performed with 20 ng of bacterial DNA. First, a multiplex PCR reaction for the detection of *arpA*, *chuA*, *yjaA* and TspE4.C2 was performed. In some cases, additional PCR reactions were performed to assign the strains to phylogroups C and E. For these cases, the primers ArpAgpE.f and ArpAgpE.r were used for phylogroup E and primers trpAgpC.1 and trpAgpC.2 were used for the detection of phylogroup C. After PCR, 5 µL of PCR products were resolved by 1.5% agarose gel electrophoresis (0.5xTBE) at 100 V for approximately 1 h. Then the gels were stained with ethidium bromide and visualized with the GelDoc (Biorad) system as for DNA fingerprinting. The strains were assigned to 7 phylogroups: A, B1, B2, C, D, E and Unknown.

### 2.6. Detection of the bla_CTX-M Gene Fragment by PCR

For detection of the bla_CTX-M gene fragment, PCR reactions were performed with the use of qCTX-M-F and qCTX-M-R primers, as published by Wang et al. (2022) [31]. The primers were used in standard 25 µL PCR reactions as follows: 1× Taq buffer with MgCl_2_; 0.4 µM of each primer; 1U of the Taq Polymerase (Thermo Fisher Scientific); 80 µM of dNTPs; and 10 ng of bacterial DNA. The procedure for PCR amplification included an initial denaturation step at 95 °C for 5 min; 34 cycles of denaturation at 95 °C for 10 s, a primer annealing step at 60 °C for 30 s and an elongation step at 72 °C for 30 s. The PCR products were resolved using 2% agarose gel electrophoresis in 0.5× TBE at 100 V for 45 min. Gels were then stained with GelRed (Thermo Fisher Scientific) and visualized using the GelDoc (Biorad) documentation system.

### 2.7. Antimicrobial Susceptibility Testing

The double-disk synergy (DDS) assay was carried out to identify ESBL-producing strains [32]. The *E. coli* isolates were tested using a Kirby–Bauer disk diffusion assay for susceptibility to the following 16 antimicrobial agents, according to the European Committee for Antimicrobial Susceptibility Testing (EUCAST) v.13.1 (2023) guidelines (The European Committee on Antimicrobial Susceptibility Testing. Breakpoint tables for interpretation of MICs and zone diameters. Version 13.1, 2023. https://www.eucast.org (accessed on 3 March 2025)): AMP (ampicillin 10 µg), AMC (amoxicillin/clavulanic acid 10/30 µg), TZP (piperacillin/tazobactam 30/6 µg), CXM (cefuroxime 30 µg), CAZ (ceftazidime 10 µg), FEP (cefepime 30 µg), CTX (cefotaxime 5 µg), CIP (ciprofloxacin 5 µg), CN (gentamicin 10 µg), AK (amikacin 10 µg), IPM (imipenem 10 µg), MEM (meropenem 10 µg), ETP (ertapenem 10 µg), TGC (tigecycline 15 µg), SXT (sulfamethoxazole/trimethoprim 25/25 µg), and FOX (cefoxitin 30 µg) (Oxoid). *E. coli* ATCC 25922 was used as a reference strain. The QC control table, adapted from EUCAST QC Tables v 13.1, was incorporated into Table S2. The tests were performed with the use of standard 9 cm wide Petri dishes (Sarstedt, Nümbrecht, Germany) filled with 20 mL of Mueller–Hinton medium (Difco, Franklin Lakes, NJ, USA). Bacterial cultures were resuspended in sterile 0.85% NaCl to reach 0.5 McFarland and spread on Mueller–Hinton medium. Then, the plates with up to 6 antibiotic disks put on the medium surface were incubated for 18–24 h at 37 °C. Finally, the diameters of the growth inhibition zones were measured. The experiment was performed at least two times.

### 2.8. Data Analysis

Principal component analysis (PCA) was performed with the use of PAST 5 software [33]. PCA included analysis of 16 features (standardized Z-scores of the raw data on average diameters measured in the antibiotic resistance assay; Table S2) for 114 *E. coli* strains. Data analysis and visualization were performed in the R environment using the dplyr, pheatmap, ggplot2, and FSA packages. Statistical analysis comparing the inhibition zone diameters across phylogroups, sources and seasons was conducted using the Kruskal–Wallis test followed by Dunn’s post hoc test with Bonferroni correction, *p* < 0.05. Cluster analysis was performed using Ward’s method, with squared Euclidean distances. Hierarchical clustering was performed using a Pearson-based distance matrix, and the resulting dendrogram was constructed using the UPGMA method implemented in the ape package (R). The tree was generated on the basis of profiles obtained from sequence-based rep-PCR analysis using ERIC and BOX primers. The map was created using the online tool Datawrapper; https://www.datawrapper.de; accessed on 8 May 2025.

## 3. Results

### 3.1. Diversity of Lactose-Fermenting Isolates from the Marine Environment

**Figure 2:**
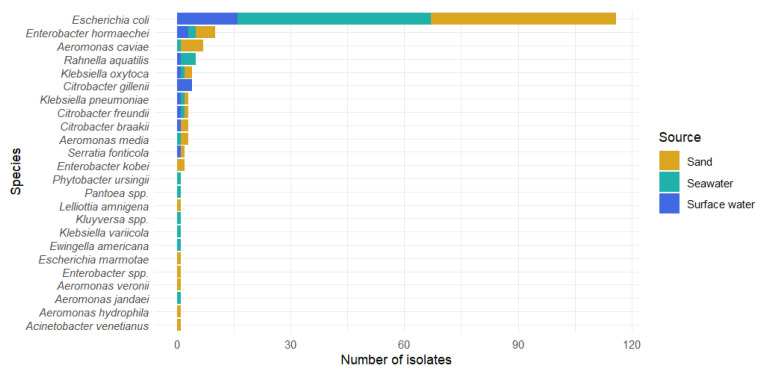
Number of strains isolated from seawater (blue), surface water (violet), and sand (yellow), along the Gulf of Gdansk (Poland), which were identified to the species or genus level by MALDI-TOF MS (n = 184; 12 strains could not be identified with MALDI-TOF MS).

Altogether, 69 samples were collected from 16 geographical locations (Figure 1), yielding 184 lactose-fermenting pure bacterial isolates. For 11 samples, no growth of lactose-fermenting bacteria was observed (Table 1). Out of these 184 strains, 75 were isolated from seawater, 32 were isolated from the surface water of rivers or streams, and 79 were isolated from beach sand (Table S1). The largest number of isolates was obtained in August 2022 (42 strains), while the smallest number was obtained in February 2023 (6 strains). The isolates were analyzed by MALDI-TOF MS for strain identification. Twelve isolates could not be identified to the species level. Nevertheless, 24 different species/genera were identified, with the most numerous being *Escherichia coli* (116 strains), followed by *Enterobacter hormaechei* (10 strains) (Table 1; Figure 2). Five of the identified species were present in all tested environments (seawater, surface water, sand), while other species were only found in one location (Figure 2). Initial screening recovered similar numbers of lactose-fermenting bacteria per sample. However, species-specific identification revealed that only 63% of the isolates were *E. coli*, resulting in a highly uneven distribution of confirmed strains across locations. This underscores that the post-isolation identification step heavily influenced the final site-specific *E. coli* recovery rates.

### 3.2. Metabolic Biodiversity of E. coli Populations Collected from Recreational Areas

Since *E. coli* isolates were the most commonly found lactose-fermenting bacteria in all three environments, their biodiversity on multiple levels was assessed. Two *E. coli* isolates were excluded from further studies because metabolic profiles were not obtained for them. A similar number of *E. coli* isolates were obtained (49 and 48, respectively) from both seawater and sand samples, while 17 *E. coli* isolates were collected from surface water sources (Table S2). Indeed, the majority of isolates from seawater and sand samples were identified as *E. coli* (65% and 60%, respectively), while from surface water, only 53% of the isolated strains were found to be *E. coli*. Altogether, the variability of features between all isolates was very high on both the genomic and metabolic levels. In the present study, as many as 25 metabolic profiles were obtained for 114 *E. coli* isolates tested using the 20E API identification kit (Biomerieux, France) (Table S2). Most of the profiles were characterized as “good” identification for *E. coli* species. The most prevalent were profiles 13 and 14, which were characteristic of 42 and 17 strains, respectively (Table S2), which differed only in their ability to use rhamnose as a sole carbon source. Another 12 strains (profile 10) were similar to profile 13; however, they presented no ornithine decarboxylase activity. Interestingly, some *E. coli* isolates presented an unusual metabolic API profile, and thus they were reanalyzed by MALDI-TOF MS, and three of the isolates (31, 34, 197) were excluded because they were not identified as *E. coli* (Figure S1). Interestingly, eight isolates (6, 7, 28, 127, 216, 219, 227, 231) categorized as profile 2 did not display lysine decarboxylase activity, which is not typical for *E. coli* strains; however, they were validated twice by MALDI-TOF MS with a good identification score. For strains isolated from three different environments, the most prevalent profile found in sand and seawater was profile 13 (approximately 40% of the strains), unlike in surface waters, where the distribution of metabolic profiles was more balanced (profiles 2, 10, 13, 14 comprised 76% of the strains). Interestingly, as many as 15 *E. coli* strains presented unique API profiles.

### 3.3. Antibiotic Susceptibility of E. coli Populations Collected from Recreational Areas

**Figure 3:**
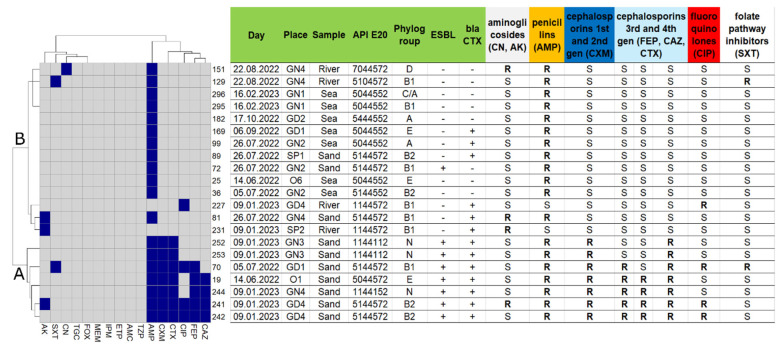
Associations between *E. coli* strains (n = 21) that were resistant to at least one antibiotic. Gray squares—susceptible, Blue squares—resistant. Aminoglycosides: AK (amikacin), CN (gentamicin); penicillins: AMP (ampicillin); cephalosporins: CXM (cefuroxime), CAZ (ceftazidime), FEP (cefepime), CTX (cefotaxime); fluoroquinolones: CIP (ciprofloxacin); folate pathway inhibitors: SXT (sulfamethoxazole/trimethoprim). GD1—Gdansk Brzezno, GD2—Gdansk Jelitkowo 66, GD4—Gdansk Oliwski Stream, GN1—Gdynia city beach, GN2—Gdynia Orlowo, GN3—Gdynia Orlowo pond, GN4—Gdynia Kacza river, SP1—Sopot, SP2—Swelina river, O1—Hel, O6—Wladyslawowo; Phylogroup N- Unknown; R—resistant, S—susceptible.

In addition to metabolic profiling, the 114 *E. coli* isolates were also evaluated for resistance to 16 different antibiotics according to EUCAST protocols (Table 2, Table S2). Within this group, all *E. coli* isolates were sensitive to seven antibiotics: a glycylcydine, a folate pathway inhibitor, three carbapenems, a penicillin + beta-lactamase inhibitor, and an anti-pseudomonal penicillin + beta-lactamase inhibitor (Figure 3; Table 2 and Table S2). However, 21 of the 114 strains (18%) exhibited resistance to at least one of the tested antibiotics (Figure 3; Table S2) and eight of them were positive for the presence of EBSL. Of these, seven *E. coli* strains were isolated from seawater, ten from sand, and four from surface water sources. Antibiotic resistance was observed in strains isolated from both water and sand; however, in sand, more strains with resistance to cephalosporins (CXM, CAZ, FEP, CTX) and fluoroquinolones (CIP) were identified (Figure 3). The largest number of strains were resistant to penicillins, corresponding to 17% of the tested isolates. For the other antibiotics, resistance levels were similar and ranged from 1 to 6%, with the highest levels observed for the cephalosporins CXM and CTX (Table 2).

As many as 7 (6.14%) ESBL-positive *E. coli* isolates were multi-drug-resistant (MDR), and all of these were originally isolated from sand (Figure 3; Table S2). The MDR isolates were defined according to their resistance to antibiotic groups, as proposed by Magiorakos et al. (2011) [34]. Although one isolate (72) only exhibited resistance to AMP, it was still ESBL-positive in the Double-Disk Synergy test. Interestingly, three of the MDRs were isolated from sand located near two rivers/streams (GN4 and GD4). The metabolic profiles of the isolated MDRs were variable representing four different API profiles (Figure 3; Table S2). Out of all isolates, 39 tested positive for the presence of the bla_CTX-M gene, and 26 of these were susceptible to all 16 tested antibiotics (Table S2). Among *E. coli* isolates that were resistant to at least 1 antibiotic, 13 strains (including all MDR isolates) tested positive for the bla_CTX-M gene (Figure 3). Interestingly, for four *E. coli* isolates within this group (81, 89, 99, 169), the presence of the bla_CTX-M gene was confirmed together with AMP resistance, while the other two isolates (227 and 231) were susceptible to penicillins and cephalosporins, despite the fact that a specific PCR product for bla_CTX-M was observed. The *E. coli* isolates formed two main clades (A and B) when grouped according to the number of antibiotics they are resistant to (Figure 3). Moreover, the seven MDR isolates placed in clade A were resistant to as many as 3–7 antibiotics from six different groups of antibiotics. Clade B was subdivided into two subclades, one of which contained 3 isolates (81, 227, 231), while the other was composed of 11 isolates that were mainly resistant to penicillins (AMP).

### 3.4. Genotypic Variability of E. coli Populations Collected from Recreational Areas

The significant variability in the metabolic profiles of the *E. coli* isolates is consistent with differences observed in their genetic repetitive sequence profiles, based on screening using ERIC and BOX primers. Based on these analyses, the isolates could be grouped into 11 (ERIC) and 7 (BOX) clades (Figures S2–S4). The 114 *E. coli* strains exhibited a high level of biodiversity, presenting many unique profiles for ERIC and BOX repetitive sequences, respectively. Isolates that exhibited resistance to at least one antibiotic were scattered along the clades in both ERIC and BOX analyses. Moreover, all of the isolates clustered differently for ERIC vs. BOX analyses, suggesting that more than one repetitive sequence in these bacterial genomes of environmental origin should be analyzed. However, in both analyses, most of the MDR *E. coli* isolates (apart from 19 and 70) were localized in one separate clade (clade 5 for BOX, clade 9 for ERIC), which was different from the majority of the isolates, suggesting there might be major differences between their profiles and the profiles of the other non-MDR *E. coli* isolates (Figures S3 and S4).

### 3.5. Correlation Between Genetic Profiles, Phylogroups and Antibiotic Resistance in the E. coli Isolates

Based on the presence of different virulence genes, the *E. coli* strains identified in the present study can be assigned to different phylogroups (A, B1, B2, C, D, E) as proposed by Clermont et al. (2013) [29]. Strains falling into groups A or B1 are primarily associated with environmental/commensal *E. coli* isolates, while strains assigned to groups B2 or D could potentially be pathogenic. Isolates belonging to phylogroups C, E, or Unknown are not primarily associated with increased or decreased potential for pathogenicity. Although most of the isolated *E. coli* (61%) were assigned to B1 and A groups, as many as 20% belonged to either B2 or D groups. However, the distribution of these two groups of isolates among the three described environments where samples were collected was not even. The majority of the isolates from surface waters belonged either to A or B1 phylogroups (76%), compared with only 54% of the isolates from seawater (Figure S5). In contrast, although none of the surface water isolates belonged to B2, seven isolates from sand were assigned to the B2 phylogroup. Most of the *E. coli* isolates that were resistant to at least one of the tested antibiotics (9 out of 21) belonged to commensal groups (A and B1), while only five were assigned to more pathogenic groups (B2, D) (Figure 3). Comparison of phylogroup assignments with ERIC and BOX clades shows that the assigned phylogroups are scattered along different clades for both analyses, with no clear distinction or grouping by phylogroups, nor by place or date of collection (Figures S3 and S4; Table S2). For the 21 *E. coli* isolates resistant to at least one antibiotic (Figure 3, Table S2), the variability in their ERIC and BOX profiles was high. Most of the strains exhibited unique profiles, falling into seven different ERIC clades and five different BOX clades (Table S2). The strains were isolated from 10 different locations over eight collection campaigns.

**Figure 4:**
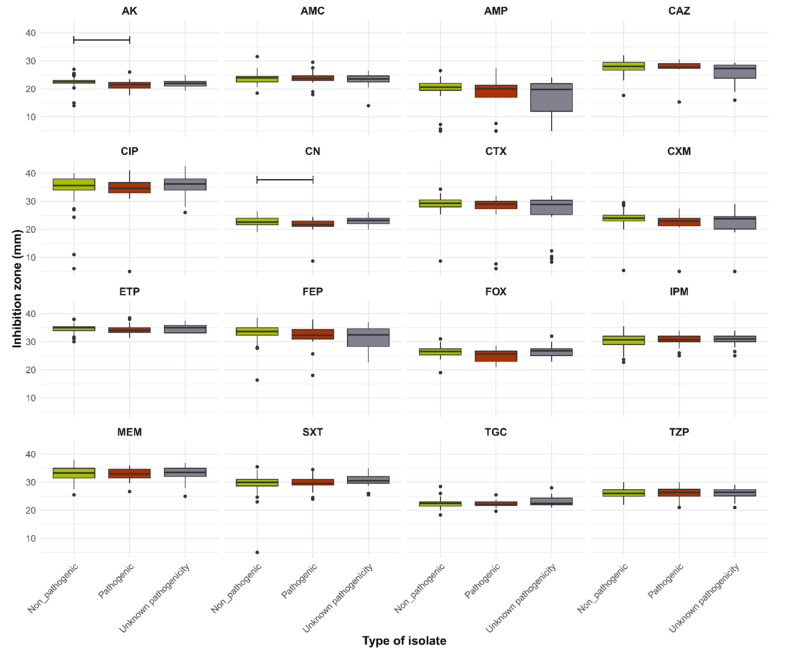
Average growth inhibition zone diameters in antibiotic susceptibility assays for *E. coli* strains grouped by phylogroup. Strains were grouped according to phylogroup (non-pathogenic—phylogroups A and B1 (n = 69), pathogenic—phylogroups B2 and D (n = 22); unknown pathogenicity—phylogroups C, E and unknown (n = 16). AK—amikacin; AMC—amoxicillin-clavulanic acid, AMP—ampicillin, CAZ—ceftazidime; CIP—ciprofloxacin; CN—gentamicin; CTX—cefotaxime; CXM—cefuroxime; ETP—ertapenem; FEP—cefepime; FOX—cefoxitin; IPM—imipenem; MEM—meropenem; SXT—trimethoprim-sulfamethoxazole; TGC—tigecycline; TZP—piperacillin-tazobactam. Statistically significantly different results are shown with black lines, after Kruskal–Wallis rank sum analysis test with Dunn’s post hoc test, at *p* < 0.05.

Interestingly, average inhibition zone diameters for phylogroups A and B2 obtained by antibiotic resistance analysis were significantly smaller for AK, CN and CXM in B2 than in A (*p* = 0.0007; *p* = 0.0091 and *p* = 0.0228, respectively); while for AMC significant differences were observed between average growth inhibition zone diameters between phylogroups A and B1 (*p* = 0.0324) (Figure S6). Moreover, based on comparison of average growth inhibition zone diameters between pathogenic (B2, D), non-pathogenic (A, B1), and unknown pathogenicity (C, E, Unknown) *E. coli* groups, the diameter of growth inhibition was significantly lower for the pathogenic group than the non-pathogenic group for AK and CN (*p* = 0.0064; *p* = 0.0254, respectively) (Figure 4).

**Figure 5:**
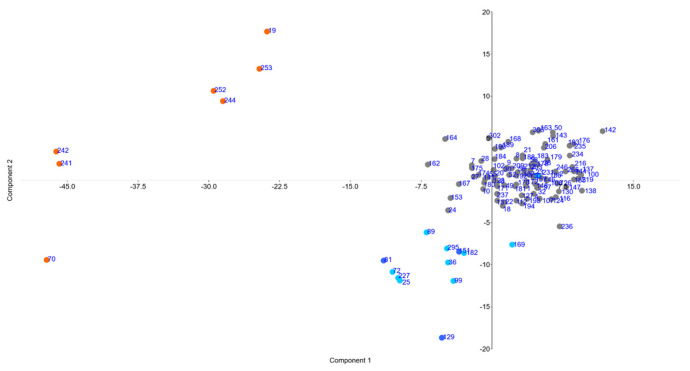
Principal component analysis of 16 features of the 114 *E. coli* strains. PCA included 16 results (average diameters of inhibition zones) from antibiotic resistance testing. The analysis was performed using Past 5 software [33]. Strains resistant to none (gray), one (light blue), two (blue), or at least three (orange) groups of antibiotics are marked with different colors. PC1 = 48.8%; PC2 = 11.6%.

To investigate possible phenotypic connections among the population of *E. coli* isolated from the Gulf of Gdansk, principal component analysis was employed and fed with the z-scores of average diameters of growth inhibition zones measured for each of the 16 tested antibiotics (Figure 5). Use of inhibition zone diameters has been previously reported to assess the population susceptibility and distribution of bovine mastitis pathogens in Belgium [35]. First, the PC1 and PC2 components were able to explain as much as 48.8% and 11.6% of the variability between the strains. Based on these analyses, z-scores of the average diameters of growth inhibition zones for aminoglycoside (CN), cephalosporins (CTX), antipseudomonal penicillin + beta lactamase inhibitors (TZP) and carbapenems (MEM) (loadings for PC1 ~0.45) were the most influential factors. Principal component analysis also suggests that 7 MDR strains belong to a separate clade, which is further divided into two subgroups (Figure 5). Interestingly, *E. coli* strains 244, 252, and 253, which were isolated in GN on 9 January 2023, were all categorized into the Unknown phylogroup and clustered together in the same ERIC clade, unlike isolates 19 and 70. Other isolates that were resistant to one or two groups of antibiotics clustered in the same area, forming a large clade. Although they were closely grouped near each other, the whole clade was also positioned close to the majority of the strains tested, the susceptible ones. Interestingly, isolate 231, which is resistant to one of the aminoglycosides, was grouped together with the susceptible *E. coli* strains. Moreover, it seems that the use of average diameters of growth inhibition zones could enable more detailed insight into the spatial distribution of strains along the tested features. For example, susceptible isolates 10, 24, 153, 162, 164, and 167 seem to be more similar to strains resistant to one or two groups of antibiotics along the PC1 axes than to isolates susceptible to all antibiotics tested. Presumably, it is possible that other features could also influence the population dynamics of this group of *E. coli* isolates.

### 3.6. The Possible Impact of the Environment on Drug-Resistant E. coli Occurrence in Recreational Areas

The biodiversity of *E. coli* isolates found in the marine environment may be affected by specific environmental factors, such as salinity, seasonal variations or proximity to chemical and other contamination sources. The Baltic Sea is a brackish sea with low salinity, and salinity levels in the Gulf of Gdansk were similar in all places tested, including seawater and sand from the intertidal zone. Moreover, while salinity levels are lower at the mouth of the Vistula River, the largest river that drains into the Gulf of Gdansk, than in the surrounding waters, this location was not sampled. Thus, salinity levels were assumed to be approximately constant for all samples and are not expected to be an influential factor on the diversity or survival of *E. coli* strains identified in the Gulf of Gdansk in the present study. The number of drug-resistant *E. coli* that were isolated from different environments was also unevenly distributed. Eight out of 21 *E. coli* isolates that were resistant to at least one antibiotic were obtained from water or sand near rivers or streams (GD4, GN4, and SP2). However, most of the isolates came from sand surrounding a surface water source, rather than from the surface water itself (Figure 3, Table S2).

**Figure 6:**
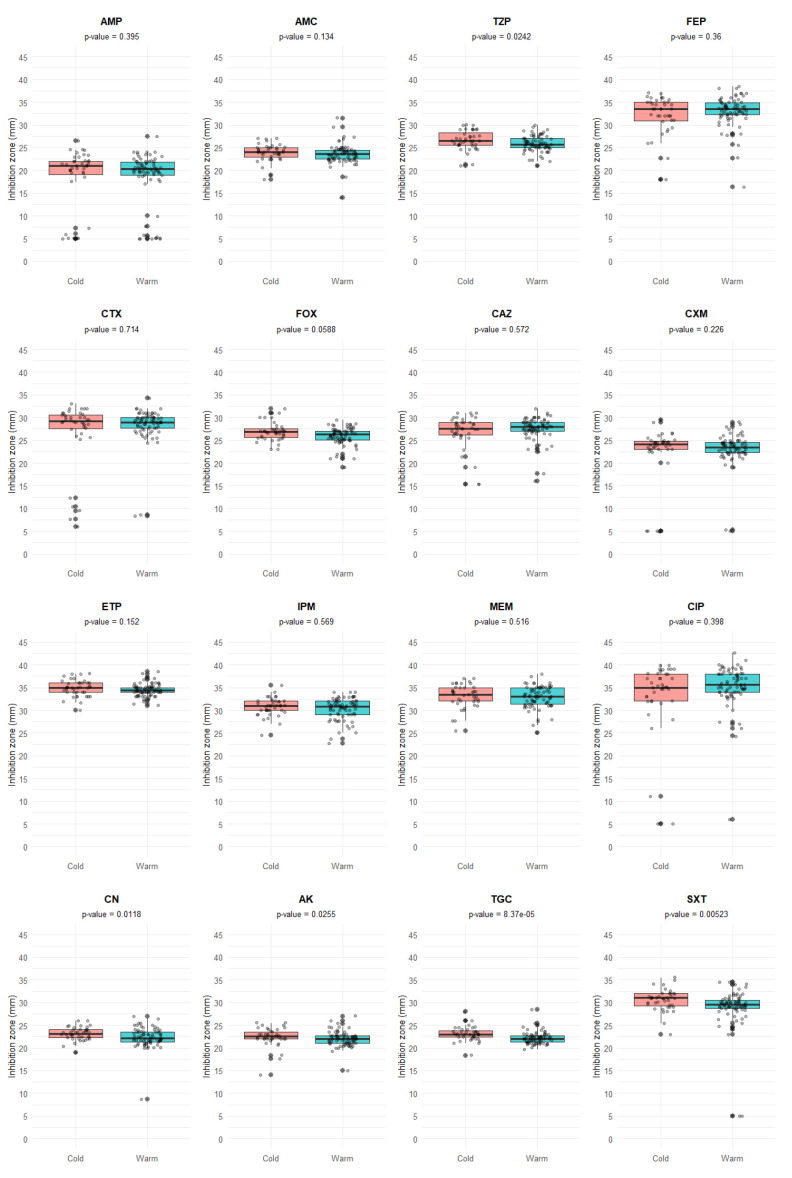
Average growth inhibition zone diameters according to seasonal temperature as measured in antibiotic susceptibility assays. *E. coli* strains were grouped accordingly to seasonal temperature (Warm—June, July, August, September (n = 74), Cold—October, January, February (n = 40). AK—amikacin; AMC—amoxicillin-clavulanic acid, AMP—ampicillin, CAZ—ceftazidime; CIP—ciprofloxacin; CN—gentamicin; CTX—cefotaxime; CXM—cefuroxime; ETP—ertapenem; FEP—cefepime; FOX—cefoxitin; IPM—imipenem; MEM—meropenem; SXT—trimethoprim-sulfamethoxazole; TGC—tigecycline; TZP—piperacillin-tazobactam. Statistical significance of comparisons is shown, after Kruskal–Wallis rank sum analysis test with Dunn’s post hoc test, at *p* < 0.05.

In different seasons, environmental conditions, such as higher or lower temperatures, may favor the presence of different numbers or strains of bacteria in marine waters, as well as their overall survival time. Also, the influx of contaminated water is usually greater during the warmer, summer recreational season than in colder months. Indeed, in the Gulf of Gdansk, variations in average growth inhibition zone diameters were observed between *E. coli* isolates collected in colder (autumn, winter) vs. warmer seasons (spring, summer) (Figure 6). Interestingly, for strains collected in the warmer months, average growth inhibition zone diameters were found to be significantly lower for six antibiotics tested, compared to strains collected in the cold season. Three of these antibiotics (aminoglycosides: AK, CN; folate pathway inhibitor: SXT) are classified in the Access WHO AWaRe group of antibiotics, while others (antipseudomonal penicillins + beta-lactamase inhibitors: TZP; cephamycin: FOX and glicylcidyne: TGC) belong to either the Watch or Reserve group.

## 4. Discussion

The Baltic Sea is a shallow brackish sea with unique environmental and geographical characteristics that shape the biodiversity of organisms which preferentially live there. In particular, the salinity gradient present in the Baltic Sea may influence the survival of microorganisms and could affect their geographical distribution based on tolerance to local salt concentrations [1]. Moreover, the Baltic Sea marine environment has been shown to be relatively contaminated with pharmaceutical compounds, including antibiotics [5,9,10,11,12]. These antibiotics could create selective pressure, which encourages the survival and growth of antibiotic-resistant microorganisms discharged into the sea from raw sewage or other sources. In turn, the presence of such antibiotic-resistant microorganisms in the marine environment may pose health risks to both wild animals and people who use recreational waters and beaches [36].

In the present study, 18% of the collected *E. coli* strains exhibited resistance to at least one of the tested antibiotics. Of these, only seven strains (6.1%) were classified as MDR and ESBL-positive, consistent with results reported for Santos Bay in Brazil (2.1%) [37]. On the other hand, in sediments collected along the Swedish Baltic Sea coast, at a depth of 5–28 m, only one *E. coli* strain (out of 30 isolated and sequenced) was found to be resistant to CN and tetracycline in an antibiotic susceptibility test [38]. However, the majority of the isolated strains did contain antibiotic resistance genes in their genomes, which were not functional under the tested conditions [38]. Indeed, similar results were obtained in our study, where the bla_CTX-M gene fragment was found to be present in 34% of the *E. coli* isolates, while 66% of these strains exhibited no resistance to antibiotics. A key explanation for this discrepancy is the variability in gene expression driven by genetic context. The expression of bla_CTX-M genes is strongly influenced by upstream regulatory elements, particularly insertion sequences such as ISEcp1, which provide promoter activity of varying strength and can significantly modulate transcription levels [39,40]. Consequently, some isolates may harbor the gene but express it at levels insufficient to exceed clinical breakpoints. In addition, the discrepancy between genotype and phenotype may reflect condition-dependent silencing of resistance genes, potentially influenced by environmental factors. A similar phenomenon was described by Enne et al. (2006), who demonstrated that intact resistance genes in *E. coli* could remain phenotypically silent under specific conditions while retaining the ability to be reactivated [41]. Comparable findings were also reported for *Klebsiella pneumoniae*, where clinically susceptible strains were shown to harbor inactive ESBL genes with intact promoter and coding regions, suggesting that apparently non-resistant isolates may still represent a latent reservoir of resistance determinants that can be activated under antibiotic pressure [42]. In addition, in another region of the Baltic Sea, Lübcke et al. (2024) found 30 *E. coli* ESBL-producing strains in a study of 124 marine water samples collected along the German coast (2021–2022), from which only 19 strains were confirmed to be MDR [5]. These data suggest that there are differences in the prevalence of ESBL-producing *E. coli* strains in the Baltic Sea, and that a significant portion of these have not yet developed into fully MDR strains. Results from the German coast of the Baltic Sea suggest that 63% of ESBL-producing isolates are MDR; whereas for the Gulf of Gdansk, we found that 75% of ESBL-producing isolates were also MDR. At the same time, antibiotic-resistant *E. coli* have been reported to be present in almost 40% of tested mammals in the Baltic Sea [22]. Thus, it seems that the prevalence of drug-resistant *E. coli* in the Baltic Sea is variable and dependent on different important factors, such as distance from the efflux pipes of wastewater treatment plants and/or proximity to urban areas, as shown by Lübcke et al. (2024) [5]. However, for the collection areas sampled in the present study, such differences were not found, possibly because the samples were collected only from recreational beaches. Possible pathways by which drug-resistant *E. coli* could enter the Gulf of Gdansk ecosystem could be from the mammals that live there [22], wastewater treatment plants [21], influents from rivers/streams and water reservoirs, or wild birds, as was shown earlier [43]. Total water inflow into the Gulf of Gdansk has been estimated to be up to 4.4 × 10^10^ m^3^ per year, where most of the water comes from the Vistula River (80% of the total inflow, Table S3). Although water inflow from wastewater treatment plants is only 0.15% of the total inflow, it may be responsible for most of the *E. coli* present in the Gulf of Gdansk. However, it has been shown that the effluents from the two biggest wastewater treatment plants at outfalls contained less than 100 CFU/100 mL of *E. coli* [21]. In addition, the Vistula River may also be a major source of *E. coli* in the Gulf of Gdansk, since many wastewater treatment plants efflux water upstream of the river mouth.

Thus, because there are many different sources of *E. coli* in marine waters, the general abundance of drug-resistant *E. coli* in a particular environment can be variable. For example, along the coast of Portugal, as many as 85% of isolated *E. coli* strains were drug-resistant, while the prevalence of drug-resistant E. coli strains in other warm climates, such as the Adriatic Sea, or Santos Bay in Brazil, was 35% and 56%, respectively [37,44,45]. In our study, the most common resistance observed among the *E. coli* strains was to penicillin class antibiotics (AMP; 18%), in agreement with results reported from the Adriatic Sea (16.5%) and Santos Bay, Brazil (20–22%), but much lower than that found in Baltic Sea mammals (48.8%) or along the Portuguese coast (33.8%) [22,37,44,45]. Interestingly, in *E. coli* strains isolated from Portugal, the Gulf of Gdansk, and Baltic Sea mammals, the prevalence of aminoglycoside-resistant *E. coli* was low (from 3% to 5%) [22,44]. Although SXT and tetracycline are two of the most often quantified and detected antibiotics in marine waters, little resistance to these antibiotics was found in our *E. coli* isolates (2% and 0%, TGC in this study). However, SXT and tetracycline resistance were much more common in other countries such as Portugal, where 23.5% and 46% of isolates were resistant to SXT or tetracycline, respectively [44]. Similarly, SXT and tetracycline-resistant strains were also more abundant in the Adriatic Sea, comprising 12% of isolates for SXT and 28.4% for tetracycline resistance [45].

With respect to phylogroup distribution patterns, a similar number of *E. coli* strains from phylogroup A were found both in Portugal and the Adriatic Sea (33–38%) [44,45], while for Baltic Sea mammals and in the Gulf of Gdansk, this number was much lower (16–18%). Interestingly, the opposite trend was observed for phylogroup B1, which was more prevalent in the Gulf of Gdansk (58%) than in Portugal or the Adriatic Sea (27–33%). The largest difference was observed for strains assigned to phylogroup B2, which was most abundant in Baltic Sea mammals (39.5%) [22], while in the Gulf of Gdansk, B2 represented only ~10% of the sampled population. Interestingly, antibiotic resistance among the *E. coli* isolates from the Gulf of Gdansk was more associated with commensal phylogroups A and B1 than with the more pathogenic B2 and D phylogroups. However, more studies are needed to better understand this phenomenon. For example, Erb et al. (2024) found that B1 isolates had greater capacity to form biofilms in a medium supplemented with NaCl than *E. coli* isolates from B2 phylogroups, which may indicate that B1 isolates are better adapted to the marine environment [38,45]. Despite differences in the distribution of phylogroup assignments, even larger biodiversity was evident based on genetic and metabolic profiling. Interestingly, for some isolates, the metabolic profiles obtained in the present study were not consistent with the standard biochemical footprint associated with *E. coli*. It may be that some of these strains belong to cryptic clades, or could represent another species, such as *E. vulneris* or *E. hermannii* [46]. However, all of these isolates were clearly identified by MALDI-TOF MS analysis. Thus, it seems that due to the inherently high variability in environmental isolates of *E. coli*, several identification methods may be required. The significant biodiversity present among the *E. coli* strains collected from the Baltic Sea was also reported for *E. coli* isolates from the Curonian Lagoon (Baltic Sea), and from Baltic Sea mammals based on XbaI digestion of their genomic DNA [22,47]. It has been shown previously that *E. coli* isolated from diverse environments present a high genotypic variability (e.g., 650 profiles, diversity not saturated) [48]. A high-level of diversity was also shown for ESBL-producing *E. coli* strains isolated from retention reservoirs in the vicinity of the Gulf of Gdansk, based on analysis of ERIC profiles [23]; however, despite the proximity of the collection points utilized, these previously published profiles were different from the ones obtained in the present study. Assessment of ERIC and BOX profiles is widely implemented to evaluate intraspecific similarity, given that identical banding profiles typically denote a common ancestry [49]. In the present study, rep-PCR was utilized to assess environmental *E. coli* diversity and monitor specific strains whose persistence across multiple locations and sampling dates would indicate prolonged environmental survival. Notably, rep-PCR profiles generally do not directly correlate with multi-locus sequence typing (MLST) or whole-genome sequencing (WGS) data [50,51]. Environmental *E. coli* strains can be characterized by their ability to survive for prolonged periods in marine environments, indicating that they could pose a higher risk for multidrug resistance dissemination. In the present study, one of the MDR isolates (70) had a BOX profile that was very similar or nearly identical to several other isolates (55, 52, 58). Hypothetically, it is possible that *E. coli* 70 may have acquired resistance genes by horizontal gene transfer, which would imply that it is more competent at acquiring exogenous DNA than non-environmental isolates, as was shown previously for *E. coli* isolated from *Daphnia* spp. [52]. It would be interesting to compare all isolates with the same BOX profile for their competence at acquiring foreign DNA in marine environmental conditions. Also, the abundance of drug-resistant *E. coli* strains was similar in both surface and seawater areas sampled in the present study. Usually, the amount of *E. coli* or allochthonous bacteria in running water is lower than in the sea; however, here this was not the case. It might be possible that in these particular places, wild birds carrying drug-resistant *E. coli* in their bodies use the banks of rivers and/or streams more often than open sea areas, suggesting that they could be the source of drug-resistant bacteria found in the sand. In support of this, a study by Rybak et al. (2022) demonstrated the presence of drug-resistant bacteria in birds caught in the vicinity of the Gulf of Gdansk [43].

In summary, in the present study, 18% of the *E. coli* strains exhibited resistance to at least one antibiotic. However, based on the diameters of resistance zones measured by the disk diffusion method, different fractions of resistant and non-resistant *E. coli* isolates could be distinguished, and a trend toward higher antibiotic resistance in the warmer months could be noticed (Figure 6). This may be particularly important, since *E. coli* isolated during warmer months exhibited significantly lower average growth inhibition zones in the presence of antibiotics not only from the Access (AK, CN, SXT) group, but also from the more critical Watch (FOX, TZP) and Reserve (TGC) WHO AWaRe groups. These data may prove to be crucial for assessing the potential risk for the spread of antibiotic resistance among *E. coli* strains in the beach sand and recreational waters. Moreover, integrating these results with data on the prevalence of antibiotic resistance genes (ARGs) in the area could provide a novel set of recommendations for robust environmental risk assessments and future monitoring strategies. Based on this study, beach sand should be integrated into routine monitoring schemes. This should be complemented by questionnaires for beachgoers, specifically assessing exposure to sand during sunbathing and recreational activities. Such measures would significantly support the evaluation and implementation of the European Union’s environmental and public health policies, including the Water Framework Directive, the Urban Wastewater Treatment Directive, the Strategic Approach to Pharmaceuticals in the Environment (2019), and the European One Health Action Plan against Antimicrobial Resistance (2017).

Future studies, such as sequencing the genomes of these isolates, would likely provide better insight into the mechanisms by which multidrug resistance is acquired in the environment at the level of microbial population. The present study represents a first step toward unlocking the key elements of *E. coli* drug resistance, persistence, and spread in the unique brackish marine environment of the Baltic Sea.

## Figures and Tables

**Table 1:** Number of lactose-fermenting isolates collected at each location and time-point, and number of resulting *E. coli* strains identified (shown in brackets). From each location, sand samples were collected. Regarding water samples, from GD3, GD4, GN4 and SP1 surface water samples were collected (river, pond, or stream), while from other collection points seawater samples were collected. nt- the samples were not collected.

City	Collection Place		Collection Coordinates	Dates of Collection											
				2022								2023			SUM
				06-14	07-05	07-26	08-02	08-09	08-23	09-06	10-18	01-09	01-11	02-16	
Gdansk	GD1	Brzezno	54.413; 18.625	nt	5 (1)	2 (2)	nt	1 (1)	6 (4)	8 (7)	4 (4)	2 (1)	nt	1 (1)	29 (21)
	GD2	Jelitkowo 66	54.424; 18.600	nt	1 (0)	0	nt	nt	4 (4)	nt	4 (4)	0	nt	nt	9 (8)
	GD3	Jelitkowo pond	54.424; 18.600	nt	1 (1)	0	nt	nt	3 (1)	nt	4 (2)	0	nt	0	8(4)
	GD4	Oliwski Stream	54.424; 18.599	nt	4 (3)	2 (0)	nt	nt	5 (3)	nt	3 (0)	9 (3)	nt	0	23 (9)
Gdynia	GN1	City beach	54.516; 18.551	nt	1 (1)	0	nt	nt	3 (3)	nt	3 (3)	6 (1)	nt	3 (3)	16 (11)
	GN2	Orlowo	54.479; 18.564	nt	2 (2)	3 (2)	nt	nt	2 (1)	nt	4 (1)	5 (1)	nt	0	16 (7)
	GN3	Orlowo pond	54.481; 18.564	nt	nt	nt	nt	nt	1 (1)	nt	nt	5 (3)	nt	nt	6 (4)
	GN4	Kacza River	54.481; 18.565	nt	3 (1)	2 (2)	nt	nt	2 (2)	nt	3 (3)	7 (5)	nt	1 (0)	18 (13)
Sopot	SP1	Sopot	54.463; 18.562	nt	1 (0)	1 (1)	nt	nt	4 (3)	nt	1 (0)	0	nt	1 (0)	8 (4)
	SP2	Swelina River	54.464; 18.561	nt	4 (2)	2 (0)	nt	nt	8 (5)	nt	7 (5)	2 (1)	nt	0	23 (13)
Other	O1	Hel	54.605; 18.801	5 (5)	nt	nt	nt	nt	nt	nt	nt	nt	0	nt	5 (5)
	O2	Jastarnia	54.694; 18.673	1 (1)	nt	nt	nt	nt	nt	nt	nt	nt	2 (1)	nt	3 (2)
	O3	Kuźnica	54.736; 18.577	3 (3)	nt	nt	nt	nt	nt	nt	nt	nt	1 (0)	nt	4 (3)
	O4	Puck	54.722; 18.416	3 (3)	nt	nt	3 (3)	nt	nt	nt	nt	nt	2 (0)	nt	8 (6)
	O5	Rzucewo	54.690; 18.469	3 (3)	nt	nt	nt	nt	nt	nt	nt	nt	nt	nt	3 (3)
	O6	Władysławowo	54.790; 18.429	3 (3)	nt	nt	nt	nt	nt	nt	nt	nt	2 (0)	nt	5 (3)
SUM				18 (18)	22 (11)	12 (7)	3 (3)	1 (1)	38 (27)	8 (7)	33 (22)	36 (15)	7 (1)	6 (4)	184 (116)

**Table 2:** Summary of resistance profiles for the tested *E. coli* isolates against 16 different antibiotics (n = 114): CN (gentamicin), AK (amikacin), AMP (ampicillin), AMC (amoxicillin/clavulanic acid), TZP (piperacillin/tazobactam), ETP (ertapenem), IPM (imipenem), MEM (meropenem), CXM (cefuroxime), FEP (cefepime), CAZ (ceftazidime), CTX (cefotaxime), FOX (cefoxitin), CIP (ciprofloxacin), TGC (tigecycline), and SXT (sulfamethoxazole/trimethoprim).

Antibiotic Groups		WHO AWaRe Group	Resistant (n = 114)	
			Number	%
aminoglycosides	CN	Access	1	0.9
	AK	Access	3	2.6
penicillins	AMP	Access	19	16.7
penicillins + beta-lactamase inhibitors	AMC	Access	0	0.0
antipseudomonal penicillins + beta-lactamase inhibitors	TZP	Watch	0	0.0
carbapenems	ETP	Watch	0	0.0
	IPM	Watch *	0	0.0
	MEM	Watch	0	0.0
cephalosporins 2nd generation	CXM	Watch	7	6.1
cephalosporins 3rd and 4th generation	FEP	Watch	5	4.4
	CAZ	Watch	4	3.5
	CTX	Watch	7	6.1
cephamycins	FOX	Watch	0	0.0
fluoroquinolones	CIP	Watch	4	3.5
glycylcydines	TGC	Reserve	0	0.0
folate pathway inhibitors	SXT	Access	2	1.8

* +cylastatin.

## Data Availability

The original contributions presented in this study are included in the article/Supplementary Materials. Further inquiries can be directed to the corresponding author.

## Supplementary Materials

The following supporting information can be downloaded at: https://www.mdpi.com/article/10.3390/microorganisms14061212/s1, Table S1. List of 186 lactose-fermenting strains isolated from marine environments sampled in the present study, along with their identification by MALDI-TOF MS. Table S2. Detailed results of metabolic activity testing with API, BOX, and ERIC profiles assignment, phylogroup assignments, and resistance patterns (for 16 antibiotics) were obtained for 114 strains identified as *E. coli*. Table S3. The main water inflows to the Gulf of Gdansk, Baltic Sea, Poland. Figure S1. Schematic representation of the re-identification workflow for the *E. coli* isolates. Figure S2. Example BOX (A) and ERIC (B) profiles for *E. coli* strains isolated from the Gulf of Gdansk between 2022 and 2023. Five microliters of PCR products were resolved by 0.8% agarose gel electrophoresis in 0.5xTBE for 2 h and 15 min, stained with ethidium bromide and visualized. A 1kb Perfect Plus DNA ladder was used as a DNA marker, and the sizes of the main DNA marker bands are labeled. Figure S3. Relationship analysis of the *E. coli* rep-PCR profiles with ERIC primers, performed in GelJ software utilizing Pearson correlation as a similarity method, and UPGMA as a linkage method with a set tolerance of 1.0. Figure S4. Relationship analysis of the *E. coli* rep-PCR profiles with BOX primers performed with GelJ software, using Pearson correlations as a similarity method, and UPGMA as a linkage method with a set tolerance of 1.0. Figure S5. Distribution of *E. coli* strains belonging to different phylogroups according to sample location (A) and environment type (B). GD1-4—Gdansk; GN1-4—Gdynia; SP1-2—Sopot; O1-6—Hel Peninsula. Figure S6. Average growth inhibition zone diameters for each E. coli phylogroup based on antibiotic susceptibility assays. Results are presented for each E. coli phylogroup (n = 15 for A; n = 54 for B1; n = 10 for B2; n = 7 for C; n = 12 for D; n = 11 for E; n = 5 for Unknown). AK—amikacin; AMC—amoxicillin-clavulanic acid; AMP—ampicillin; CAZ—ceftazidime; CIP—ciprofloxacin; CN—gentamicin; CTX—cefotaxime; CXM—cefuroxime; ETP—ertapenem; FEP—cefepime; FOX—cefoxitin; IPM—imipenem; MEM—meropenem; SXT—trimethoprim- sulfamethoxazole; TGC—tigecycline; TZP—piperacillin-tazobactam. Statistically significantly different results are shown marked with black lines, after the Kruskal-Wallis rank sum analysis test with Dunn’s post-hoc test, at *p* < 0.05. For details, see Table S1 and Table 1. References [23,53,54,55] are cited in the supplementary materials.
