# Supplementary material for: Biodiversity of Genetic, Metabolic, and Antibiotic Resistance Profiles of Escherichia coli Strains Recovered from the Baltic Sea Region
Source: Microorganisms. 2026 May 27;14(6):1212. doi: 10.3390/microorganisms14061212 (PMC13303412; doi:10.3390/microorganisms14061212)
Supplement: Supplementary file 1 [file microorganisms-14-01212-s001.zip › Supplementary Figures.pdf]

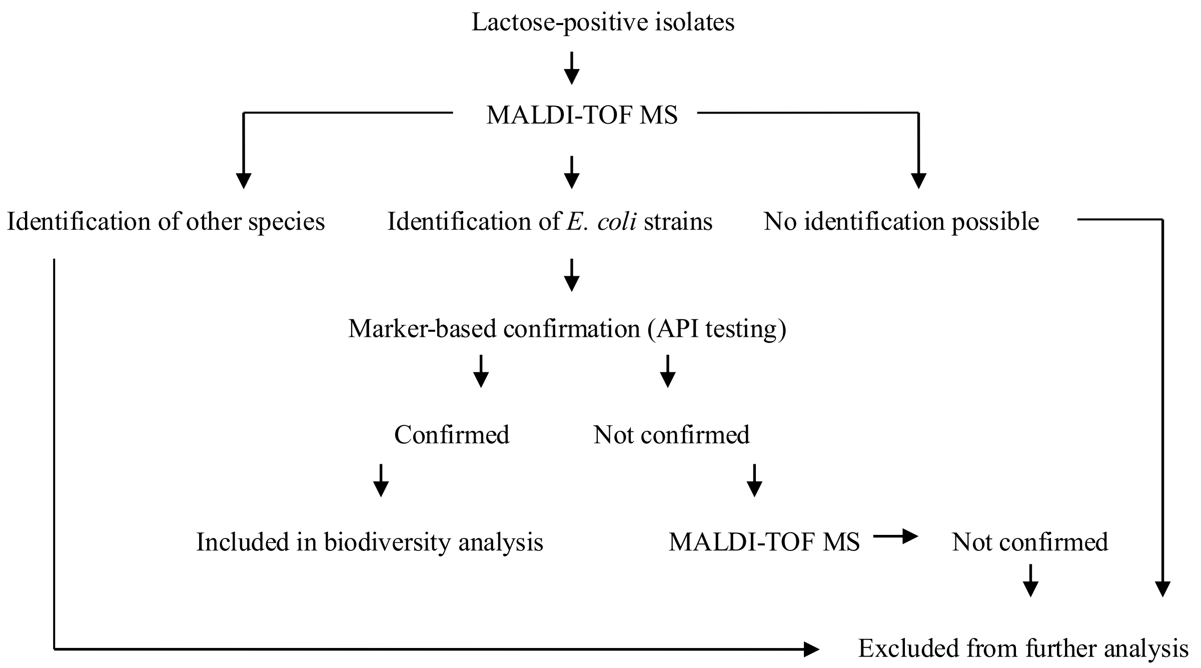

Figure S1. Schematic representation of the re-identification workflow for the *E. coli* isolates.

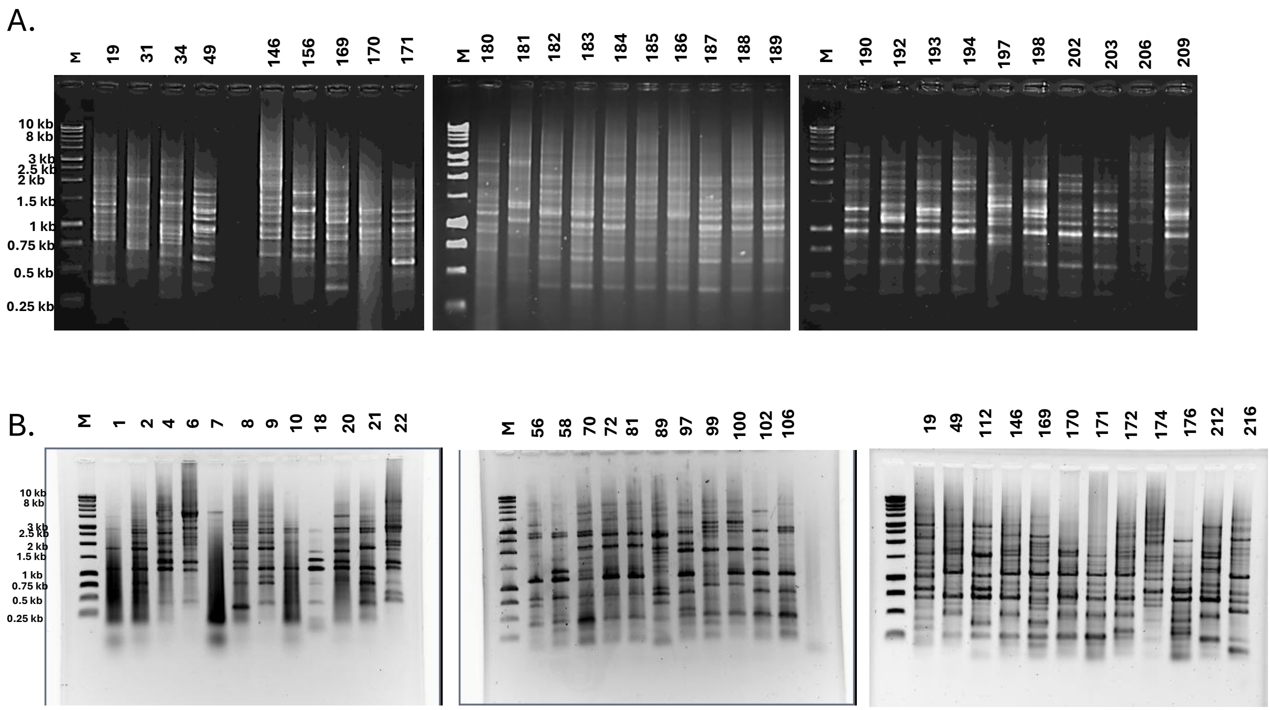

Figure S2. Example BOX (A) and ERIC (B) profiles for *E. coli* strains isolated from the Gulf of Gdansk between 2022 and 2023. Five microliters of PCR products were resolved by 0.8% agarose gel electrophoresis in 0.5xTBE for 2h and 15 min, stained with ethidium bromide and visualised. A 1kb Perfect Plus DNA ladder was used as a DNA marker, and the sizes of the main DNA marker bands are labelled.

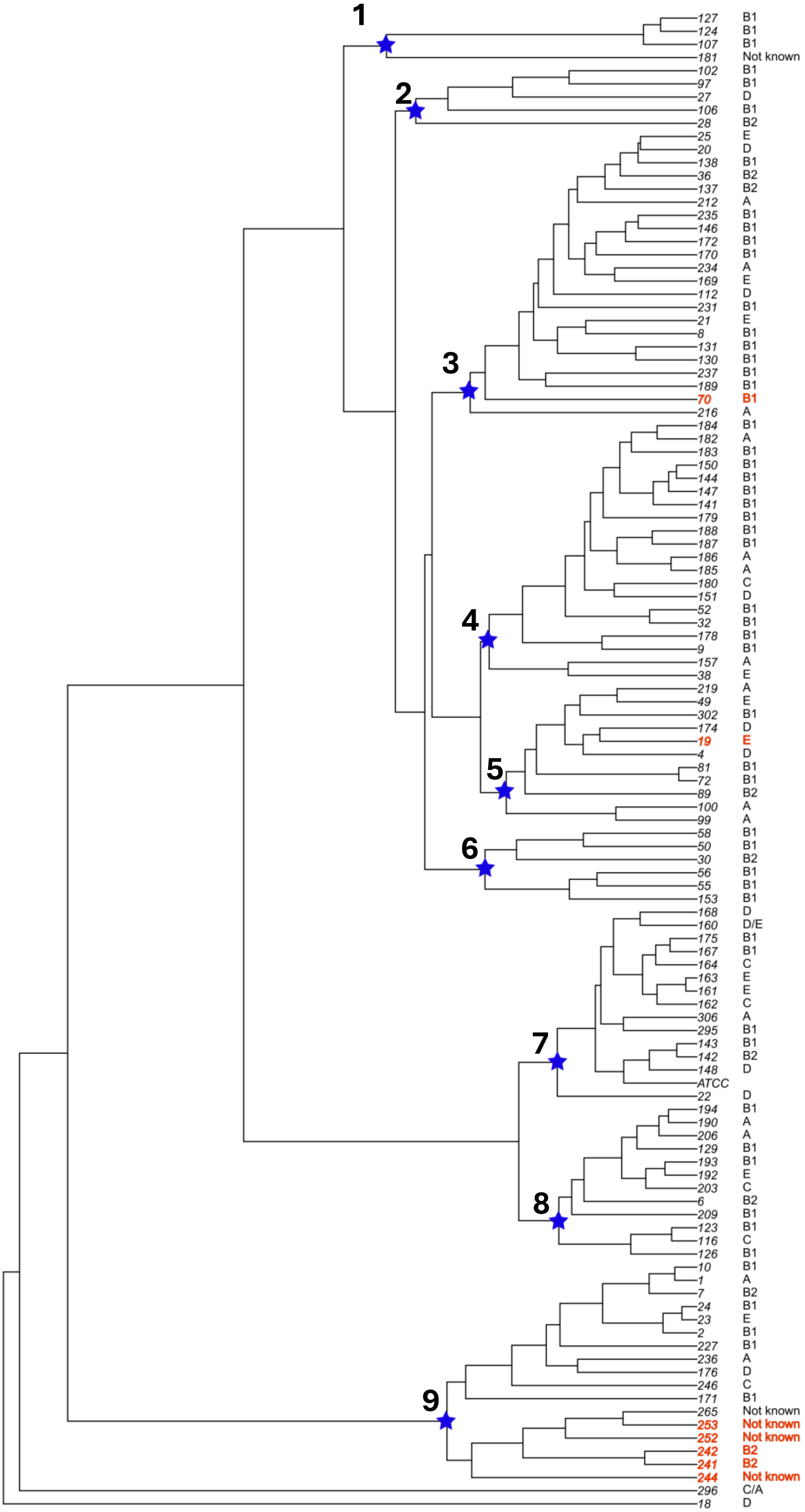

Figure S3. Relationship analysis of the *E. coli* rep-PCR profiles with ERIC primers, performed in GelJ software utilising Pearson correlation as a similarity method, and UPGMA as a linkage method with a set tolerance of 1.0.

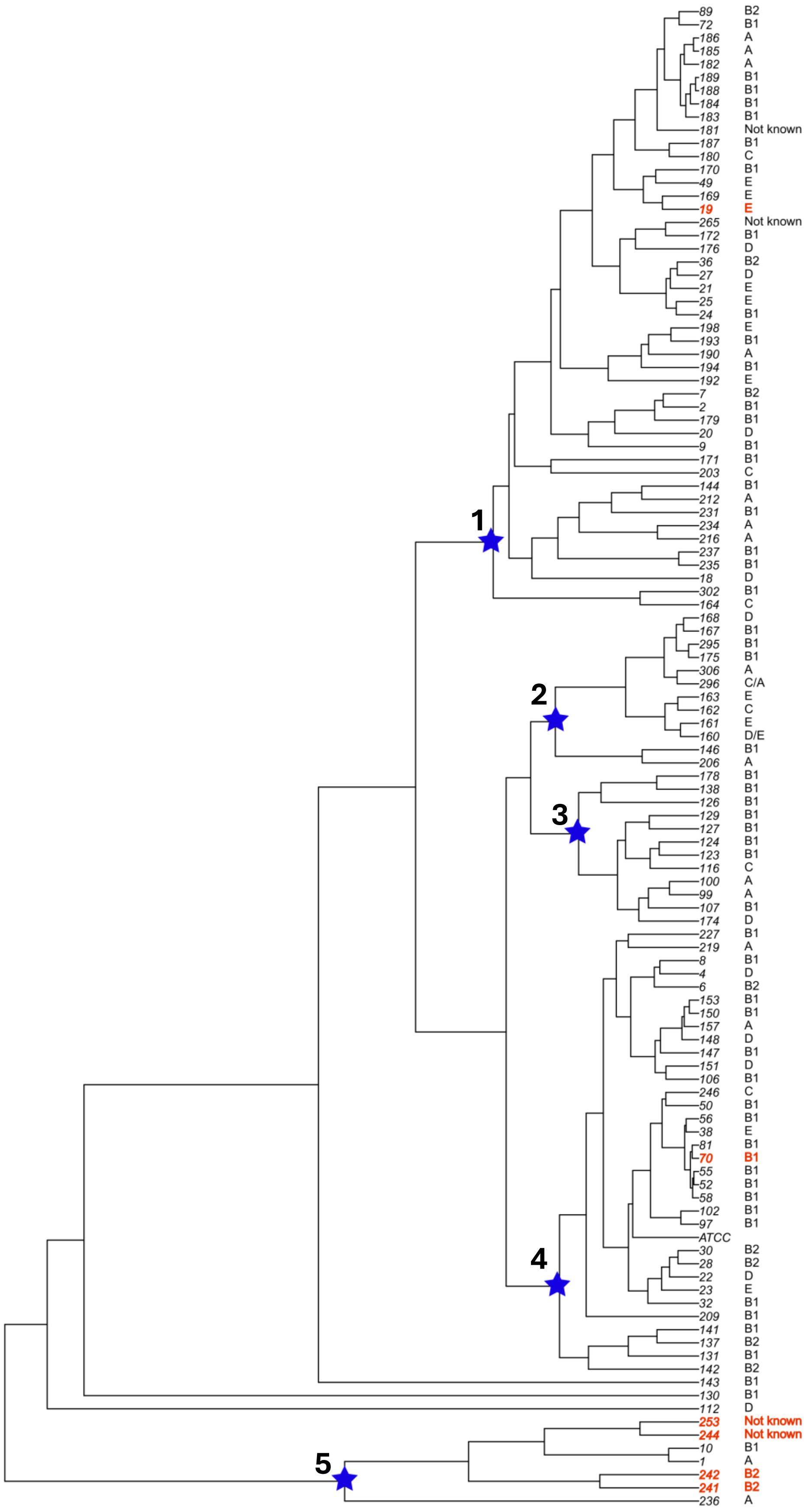

Figure S4. Relationship analysis of the *E. coli* rep-PCR profiles with BOX primers performed with GelJ software, using Pearson correlations as a similarity method, and UPGMA as a linkage method with a set tolerance of 1.0.

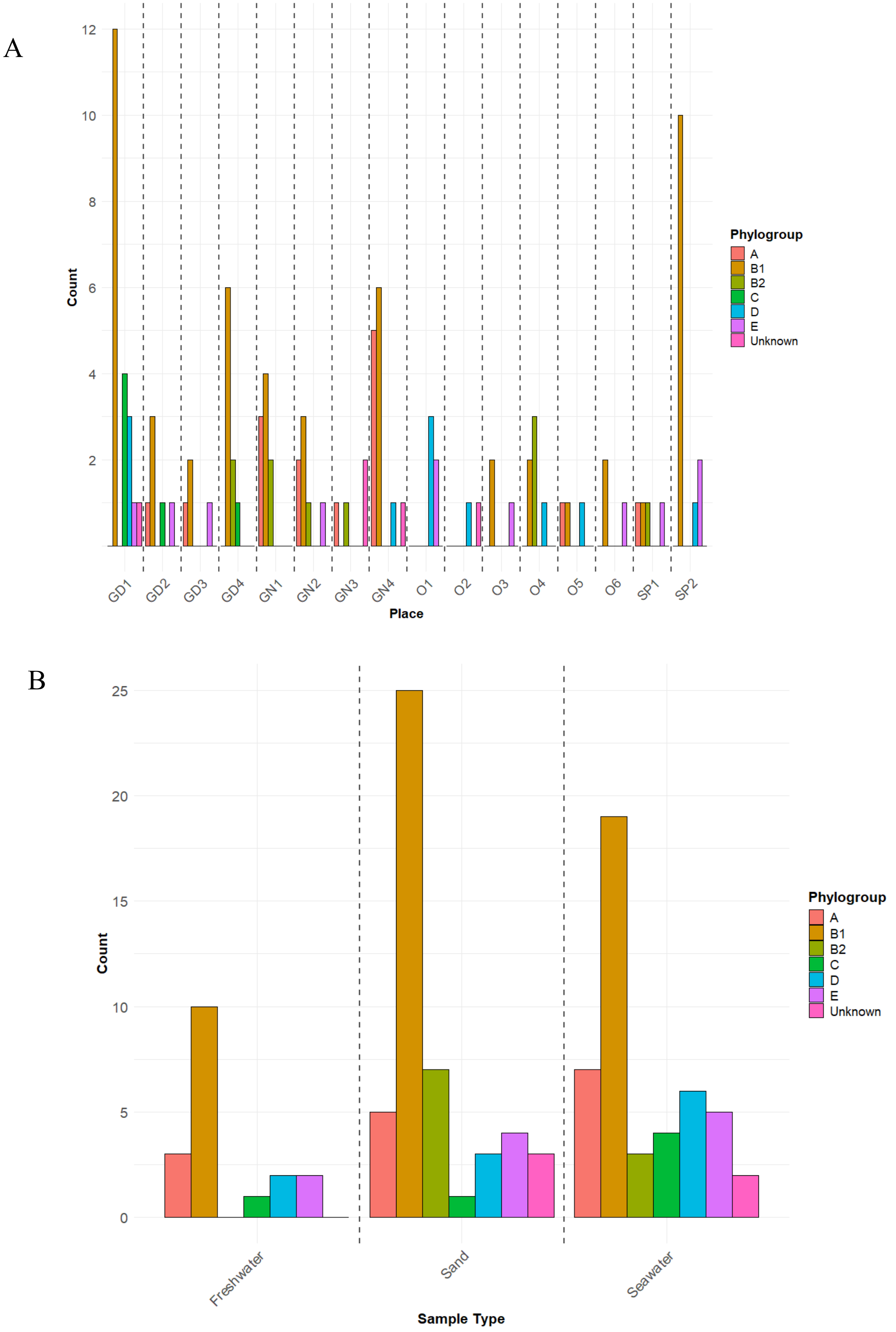

Figure S5. Distribution of *E. coli* strains belonging to different phylogroups according to sample location (A) and environment type (B). GD1-4 – Gdansk; GN1-4 – Gdynia; SP1-2 – Sopot; O1-6 – Hel Peninsula. For details, see Tab. S1 and Tab. 1.

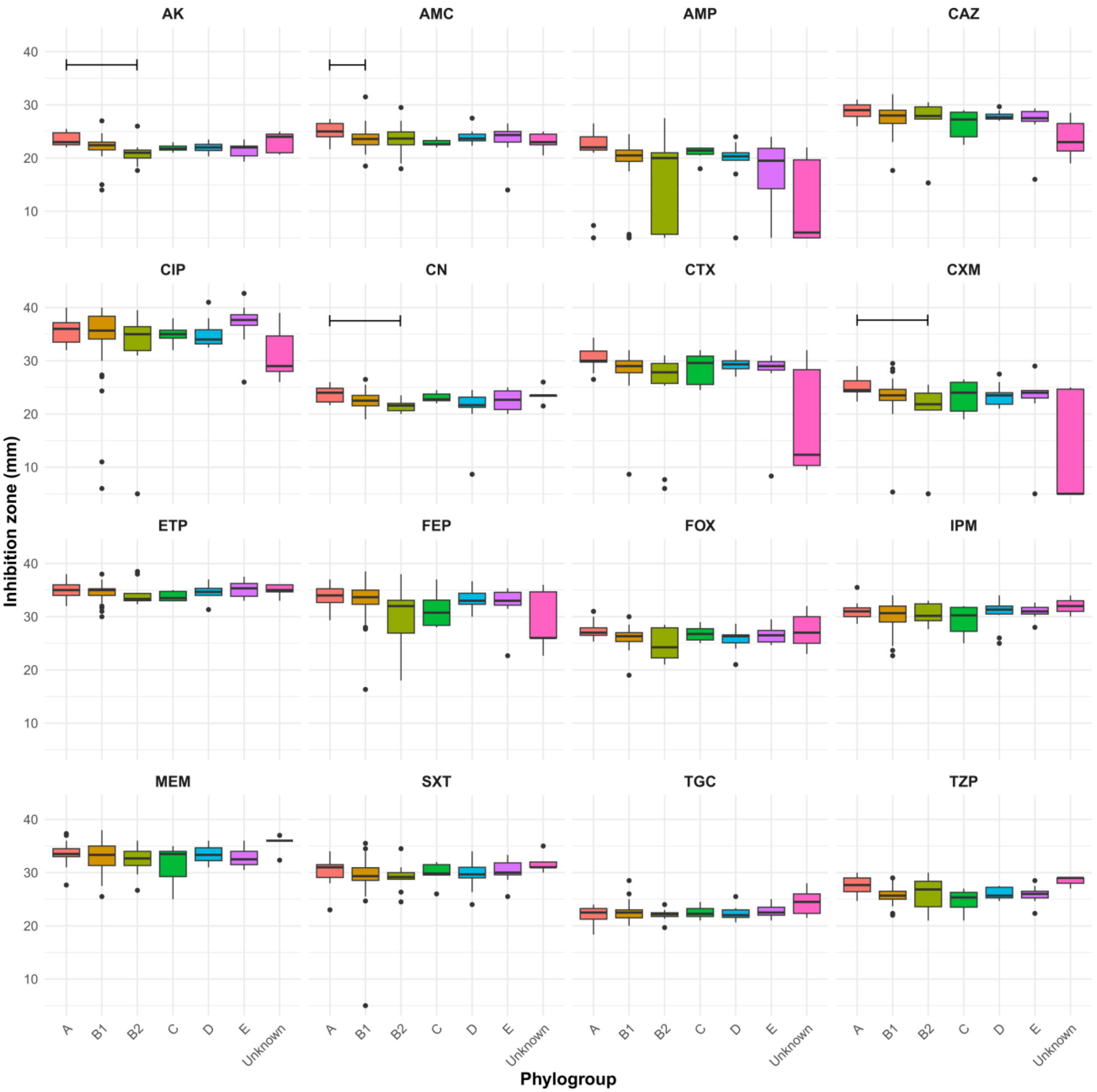

Fig. S6. Average growth inhibition zone diameters for each *E. coli* phylogroup based on antibiotic susceptibility assays. Results are presented for each *E. coli* phylogroup (n=15 for A; n=54 for B1; n=10 for B2; n=7 for C; n=12 for D; n=11 for E; n=5 for Unknown). AK – amikacin; AMC – amoxicillin-clavulanic acid; AMP – ampicillin; CAZ – ceftazidime; CIP – ciprofloxacin; CN – gentamicin; CTX – cefotaxime; CXM – cefuroxime; ETP – ertapenem; FEP – cefepime; FOX – cefoxitin; IPM – imipenem; MEM – meropenem; SXT – trimethoprim-sulfamethoxazole; TGC – tigecycline; TZP – piperacillin-tazobactam. Statistically significantly different results are shown marked with black lines, after the Kruskal-Wallis rank sum analysis test with Dunn's post-hoc test, at $p<0.05$.
